# Unusual Position of the Arteria Augnli Nasi

**Published:** 1881-06

**Authors:** Lucien Howe


### Clinical Reports.

### UNUSUAL POSITION OF THE ARTERIA ANGULI NASI.

BY DR. LUCIEN HOWE.

A brief outline of the following case shows the importance of this small vessel in certain instances, and the annoyance to which a peculiarity in its location may give rise.

The patient, Mrs. S. E., had been troubled for some years with a constant flow of tears from the left eye, due to a stricture of the nasal duct. She also suffered from a form of choroiditis with secondary cataract on the same side, but the lachrymal difficulty is the only one which interests us at present. This last had been treated with good effect, temporarily, by simply slitting the canaliculus and introducing probes of increasing size, after the time-honored method of Bowman. The stricture, however, showed a decided tendency to contract, even after having been widely dilated by a probe of large diameter, and as I had frequently observed, in such cases, a satisfactory result to follow the use of Stilling's knife, I proposed to pursue the same plan in the present instance. It will be remembered, that according to this method, a rather short, thick and almost blunt-pointed knife is passed down into the sac and through the stricture, and a probe of large size is then inserted.*

On the 14th of April, 1880, it was decided to adopt this plan of procedure and accordingly nitrous oxide gas was administered—a form of anæsthesia as invaluable in short operations about the eye as it is in dentistry or other branches of surgery.

The canaliculus was first enlarged with a probe-pointed bistouri and the Snelling's knife then passed down to the stricture. It had hardly reached this point, when a hæmorrhage begun, which, as it will be seen, forms the chief feature of interest in the case. A slight amount of bleeding was of course to be expected, nor is it a very unusual thing to divide a small twig in such a manner that the blood finds its way under the skin, producing an extensive and unsightly distension of the tissues. This, I think, has been observed by almost every operator of considerable experience. But the amount of hæmorrhage in the case under consideration, was excessive, and its continuance was probably due to the fact that the small artery had been only partially severed. For, almost immediately, an extravasation in the form of a small hard tumor the size of a walnut, showed itself over the sac. It began to spread rapidly outward and downward and upward. It could not have been more than five or six minutes before the whole lower lid was distended and discolored. The process advanced also rapidly in the upper lid, and within about ten minutes the palpebral fissure was quite

*The details of this plan and a modification in the form of the knife were described by the writer in the September number of this Journal.

firmly closed. Firm pressure over the sac did not seem to arrest the hæmorrhage, and on finding that it was impossible thus to prevent the increasing effusion into the surrounding tissues, a free and deep horizontal incision was promptly made along the centre of the lower lid. By affording an outlet thus to a part of the blood, the dangers of sloughing were much reduced. For such an event was by no means impossible, both lids being distended to their utmost limit, very hard upon pressure, the skin tense and, of course, much discolored.

In addition to the incision in the lower lid, some blood was also abstracted from the upper one, by means of a number of leeches, cold compresses were also used assiduously, and in due time the swelling subsided till at the end of some two months and a half, all traces of the swelling and even the discoloration, had disappeared. Although such an occurrence is apt to produce unnecessary alarm on the part of the patient, it is not devoid of interest to one who would consider its cause. When describing the arteria anguli nasi, Gray says, "It distributes some branches in the cheek which anastomose with the infra-orbital, and after supplying the lachrymal sac and orbicularis muscle, terminates by anastomosing with the nasal branch of the ophthalmic artery." * * * * "The student should, lastly, observe the relation of the angular artery to the lachrymal sac, and it will be seen that, as the vessel passes up along the inner margin of the orbit, it ascends on the nasal side."

Again, Merkel says,* "The two palpebral arteries, in their further course, supply the caruncula lachrymalis, the lachrymal sac with the muscles in its vicinity, and the lachrymal canal, together with the internal portion of the lids and the conjunctiva."

When we remember that the "palpebral arteries," as here described, are partly formed by anastomoses with the arteria anguli nasi, it is evident that this was the vessel wounded. The hæmorrhage from any twig of ordinary size would simply have filled the sac and caused it to overflow through the opening above.

* Handbuch der Gesammten Augenheilkunde. B. I, S. 105.

32

But, as the knife passed down, it opened the wall of the canal, and when the fluid could no longer be contained in the sac, it pressed outward, with considerable force into the loose areolar tissue, immediately adjacent. It is of interest, therefore, to see how much annoyance can be produced by a vessel which, in the normal position is hardly worthy of notice, and any such observation is not without some value, as indicating a possible complication of an operation ordinarily safe and simple.

